# Inconsistent relationships detected between seed size, shape and persistence for different plant functional groups in the Pannonian flora

**DOI:** 10.1093/aob/mcaf322

**Published:** 2025-12-12

**Authors:** Viktória Törő-Szijgyártó, Péter Török, Katalin Tóth, Hajnalka Málik-Roffa, Luis Roberto Guallichico Suntaxi, Szilvia Madar, Gergely Kovacsics-Vári, Andrea McIntosh-Buday, Patricia Díaz Cando, Judit Sonkoly

**Affiliations:** Department of Ecology, University of Debrecen, Debrecen 4032, Hungary; Department of Ecology, University of Debrecen, Debrecen 4032, Hungary; HUN-REN-UD Functional and Restoration Ecology Research Group, Debrecen 4032, Hungary; Polish Academy of Sciences, Botanical Garden – Centre for Biological Diversity Conservation in Powsin, Warszawa, PL-02973, Poland; Department of Ecology, University of Debrecen, Debrecen 4032, Hungary; HUN-REN-UD Functional and Restoration Ecology Research Group, Debrecen 4032, Hungary; Department of Ecology, University of Debrecen, Debrecen 4032, Hungary; HUN-REN-UD Functional and Restoration Ecology Research Group, Debrecen 4032, Hungary; Department of Ecology, University of Debrecen, Debrecen 4032, Hungary; Department of Ecology, University of Debrecen, Debrecen 4032, Hungary; HUN-REN-UD Functional and Restoration Ecology Research Group, Debrecen 4032, Hungary; Department of Ecology, University of Debrecen, Debrecen 4032, Hungary; Department of Ecology, University of Debrecen, Debrecen 4032, Hungary; HUN-REN-UD Functional and Restoration Ecology Research Group, Debrecen 4032, Hungary

**Keywords:** Pannonian flora, persistence, plant functional groups, seed longevity, seed mass, seed morphology, seed shape, seed size, seed weight, soil seed bank

## Abstract

**Background and Aims:**

Knowledge on seed persistence is vital from both theoretical and practical considerations but directly collecting persistence data for many species is unfeasible. Therefore, there is a need to identify traits associated with seed persistence, but studies about the effects of seed size and shape on persistence have yielded results varying across regions. We studied 392 species of the Pannonian flora (Central Europe) to assess (1) how seed mass and shape are related to seed persistence, (2) whether this relationship is consistent across plant functional groups, and (3) whether seed mass and shape are correlated in different functional groups.

**Methods:**

We collected data on the seed mass and persistence of species and performed measurements to calculate their seed shape index, with higher values indicating greater deviation from sphericity. To account for phylogenetic non-independence, we analysed how seed mass and seed shape index affect persistence in all 392 herbaceous species and separately in four plant functional groups using phylogenetic logistic regressions. To test how seed mass and shape are related to each other in these groups, we applied phylogenetic generalized least squares regression.

**Key Results:**

Across all species, both seed mass and seed shape index were negatively related to persistence, with seed mass having a stronger association. The same relationship was observed in forbs and short-lived species, but only seed shape was related to persistence in graminoid species. The relationship between seed mass and seed shape also varied between functional groups.

**Conclusions:**

Consistent with many studies in other floras, both seed mass and shape were negatively related to persistence in the Pannonian flora after accounting for phylogeny. However, only seed shape was associated with persistence in graminoid species, suggesting that different factors may be at play in forbs and graminoids. Therefore, future studies of this relationship may need to treat and analyse graminoids separately.

## INTRODUCTION

Studying soil seed banks is vital for understanding the dynamics of plant populations and communities (Hopfensperger, 2007; Plue *et al.*, 2017, 2021) and how different species deal with environmental heterogeneity and uncertainty (Long *et al.*, 2015; Gioria *et al.*, 2020). As soil seed banks disperse genetic diversity and mortality risks in time, they strongly promote the maintenance of plant populations (Gioria *et al.*, 2020). Persistent soil seed bank formation decreases the risk related to reproductive failure during periods of adverse environmental conditions, thereby constituting a bet-hedging strategy (Venable and Brown, 1988). In plant communities exposed to environmental change, for example climate change or habitat isolation, persistent soil seed banks can decrease extinction risk and contribute to population persistence (Stöcklin and Fischer, 1999 ; Estrada *et al.*, 2015). Soil seed banks also act as reserves of genetic variability (Levin, 1990; Aparicio *et al.*, 2002). As persistent soil seed banks can contain seeds produced over multiple years and years with different environmental conditions benefit different genotypes of a species, the seed bank can provide a great diversity of seed genotypes adapted to varying environmental conditions (Cabin, 1996). Therefore, persistent soil seed banks play a vital role in the resilience of plant communities (Kiss *et al.*, 2018) and the maintenance of biodiversity through space and time (Royo and Ristau, 2013). In spite of this, our knowledge on soil seed banks is still disproportionately limited compared with the above-ground vegetation.

Seed persistence is essential for the formation of a persistent soil seed bank. It refers to the ability of seeds to remain viable for a long time (Fenner and Thompson, 2005), allowing them to persist in the soil until environmental conditions are favourable, thereby promoting survival under changing or unpredictable conditions (e.g. del Cacho and Lloret, 2012; Estrada *et al.*, 2015). Seed persistence, which primarily varies on a continuous scale, is generally classified into discrete categories for simplicity and practical application. The most widespread seed bank classification system distinguishes three categories: (1) transient seeds (viable for <1 year); (2) short-term persistent seeds (viable for 1–5 years); and (3) long-term persistent seeds (viable for ≥5 years) (Thompson *et al.*, 1997). However, the boundaries are not always clear, especially between the last two categories (Thompson *et al.*, 1993). Therefore, distinguishing between transient and persistent species, without differentiating between short-term and long-term persistence, is a reasonable approach for general discussions and broad analyses (see e.g. Bekker *et al.*, 1998; Funes *et al.*, 1999; Gioria *et al.*, 2020). This distinction is especially important because it determines whether seeds of a species are able to accumulate over multiple seasons and therefore disperse in time (Baskin and Baskin, 2014).

The ability to distinguish species with transient versus persistent seeds is not only valuable for answering a wide range of fundamental questions in vegetation science and population biology (Saatkamp *et al.*, 2009), but it is crucial for the success and feasibility of restoration projects as well (von Blanckenhagen and Poschlod, 2005; Török *et al.*, 2018). Unfortunately, obtaining direct data on seed persistence is challenging and time-consuming (Cerabolini *et al.*, 2003; Jaganathan *et al.*, 2019). Soil seed bank analyses, whether using the seedling emergence or seed extraction method, require considerable effort. Moreover, the results are not necessarily conclusive, and the findings of different studies are frequently contradictory (Cerabolini *et al.*, 2003). There are other methods to assess seed longevity more accurately, but they are either costly and challenging (such as carbon-dating viable seeds, e.g. Moriuchi *et al.*, 2000), or take a particularly long time (such as burial experiments, e.g. Telewski and Zeevaart, 2002; Pakeman *et al.*, 2012). Therefore, collecting direct seed persistence data for many species is rather unrealistic. Based on the above considerations, reliably predicting the ability of seeds to persist in the soil is the only realistic option for obtaining information on the transient versus persistent nature of a wide range of species. Since such information is highly needed from both theoretical and practical conservation perspectives (e.g. Saatkamp *et al.*, 2009 ; Kalamees *et al.*, 2012), investigating which attributes correlate with seed persistence and how these attributes can improve the reliability of predictions is vitally important.

Seed mass is the most frequently measured trait of seeds (Carta *et al.*, 2024) and it is considered to have exceptionally large functional importance, as it is connected to several processes and plant characteristics, such as dispersal distance, light detection, seedling establishment, seed predation or the number of seeds produced (summarized by e.g. Moles, 2018 and Carta *et al.*, 2024). Seed shape can also influence many processes, such as soil penetration (Chambers *et al.*, 1991), fire tolerance (Ruprecht *et al.*, 2015) or the chance of surviving gut passage and therefore the potential for endozoochorous dispersal (van Leeuwen *et al.*, 2023). However, it is more challenging to quantify and consequently studied less frequently (Dayrell *et al.*, 2023; Carta *et al.*, 2024).

Seed mass and shape have been repeatedly hypothesized to be related to seed persistence, with somewhat varying results. A connection between a persistent seed bank and small, spherical seeds has already been noted by Thompson (1987). Subsequently, clear evidence for the correlation between the size, shape and persistence of seeds has been demonstrated in the British flora by Thompson *et al.* (1993): all seeds were found to be persistent within a range defined by a maximum in seed mass and seed shape variance. Since then, this relationship has been studied in the flora of other regions as well. Several such studies have confirmed that persistent seeds tend to be smaller and more spherical than transient seeds in various floras, for example in Sweden (Bakker *et al.*, 1996), Argentina (Funes *et al.*, 1999), Iran (Thompson *et al.*, 2001), Italy (Cerabolini *et al.*, 2003) and China (Zhao *et al.*, 2011). A recent study synthesizing available data for 1474 species worldwide also found that persistent seeds tend to be small and spherical (Wang *et al.*, 2024). On the other hand, Peco *et al.* (2003), for example, found that although persistent seeds tend to be smaller, seed shape is not related to persistence in Mediterranean grasslands and scrublands in Spain. Conversely, McDonald *et al.* (1996) found that while persistent seeds did tend to be more spherical, seed weight was not related to persistence in a flood meadow in Great Britain. To complicate things even further, no relationship was detected between seed size, seed shape and persistence in some other regions, such as Australia (Leishman and Westoby, 1998) or South Africa (Holmes and Newton, 2004).

The relationship between seed traits and seed persistence appears to be highly context-dependent and varies across different floras worldwide. Both factors can be influenced by a range of environmental conditions (e.g. Harel *et al.*, 2011; Abedi *et al.*, 2014; Chen *et al.*, 2021), while regional natural history and disturbance regimes further shape how variations in seed traits translate into differences in seed persistence (e.g. Leishman and Westoby, 1998). Given these complexities, it is essential to assess the relationship between seed traits and seed persistence in various regions of the world. Expanding analyses to floras with varying climates and evolutionary histories will provide a more comprehensive understanding of the strength and generality of these relationships (Leishman and Westoby, 1998).

To our knowledge, how seed size and shape are related to seed persistence has never been studied in the Central European flora, presumably due to the scarcity of seed shape data for the species of Central Europe. To facilitate the analysis of the relationship between these factors, we set out to characterize the seed shape of a large number of plant species of the Pannonian Biogeographical Region, which is located in the eastern part of Central Europe and is bordered by the Carpathians, the Alps and the Dinaric Mountains (EEA, 2016). We collected regional data on the seed mass and seed persistence of 392 species of the Pannonian flora and quantified the seed shape of all species. By analysing the compiled dataset, we aimed to answer the following questions. (1) How are seed mass and seed shape related to seed persistence? (2) Is this relationship consistent across plant functional groups? (3) Are seed mass and shape related to each other and is this relationship consistent across plant functional groups?

## MATERIALS AND METHODS

### Data collection

To analyse the relationship of seed traits and seed persistence in the Pannonian flora, we collated a dataset fully based on regionally measured data, in order to avoid the potential confounding effects of distinct climates, which can cause considerable intraspecific trait variability (Albert *et al.*, 2010; Sonkoly and Török, 2025). The Pannonian Database of Plant Traits (PADAPT; Sonkoly *et al.*, 2023) contains seed persistence data for more than 600 species based on regional soil seed bank studies. From these, we selected those species that are included in the seed collection of the Department of Ecology, University of Debrecen, and therefore available for seed shape measurements (424 species in total). In PADAPT, data on seed persistence are provided in the form of the Seed Bank Persistence Index (SBPI) following the approach of the longevity index by Bekker *et al.* (1998). The SBPI represents the proportion of data indicating the presence of a persistent seed bank for a species, ranging from 0 to 1. SBPI = 0 indicates that all available data suggest a transient seed bank for the species, while SBPI = 1 indicates that all available data suggest a persistent seed bank for the species. The thousand-seed mass (TSM) of the 424 species have already been measured on seeds stored in the aforementioned seed collection (Török *et al.*, 2013, 2016; Törő-Szijgyártó *et al.*, 2023).

### Seed shape measurements

To quantify seed shape, we measured the width, length and thickness of the seeds of all 424 species. Twenty replicate measurements were performed for each species and then all width, length and thickness data were averaged between the 20 measurements. Thickness was measured using a HEDÜ 510-201 digital thickness gauge, with an accuracy of 0.02 mm. The length and width values of the same 20 seeds were obtained from photographs using the WinImage 1.0 data acquisition system. In general, we aimed to measure diaspores in the form they are dispersed, meaning that the measured morphological unit was not necessarily a seed for all species, but here we refer to all of them as seeds for simplicity. Most grass and Asteraceae seeds were measured without appendage. *Rumex* seeds were also measured without appendage, as the presence of appendages makes accurate measurements difficult. The measured morphological unit for each species is given in Supplementary Data Table S1. The seed shape measurements were carried out on the same seed lots that were previously used for TSM measurements (see Török *et al.*, 2013, 2016; Törő-Szijgyártó *et al.*, 2023), ensuring that the measured morphological units were the same for seed mass and shape measurements.

### Data analysis

To reduce the number of confounding factors, we excluded two species groups from the analyses. We excluded aquatic plants because seed bank formation and seed persistence in aquatic habitats is presumably influenced differently by seed traits compared with terrestrial habitats. Following the approach of Powney *et al.* (2014), we categorized species with a soil moisture indicator value >8 as aquatic (based on Borhidi, 1995) and excluded eight species from the analyses based on this criterion. Trees and shrubs were also excluded from the analyses as their seed persistence seems to be influenced by seed traits differently from those of herbaceous species (Wang *et al.*, 2024) and they were represented by too few species to be analysed separately. We categorized the species into life forms based on Sonkoly *et al.* (2023), and all phanerophyte species, including nanophanerophytes (subshrubs), microphanerophytes (shrubs) and mega-mesophanerophytes (trees), altogether 24 species, were excluded from the analyses. After these exclusions, our dataset contained 392 species (Supplementary Data Table S1).

As a measure of seed shape, we calculated the seed shape index for each species. The seed shape index expresses how much the shape of a seed differs from being spherical, with a value of zero indicating a perfectly spherical seed. Increasing seed shape index values indicate increasingly flattened and/or elongated seeds. For needle- or disc-shaped seeds, the maximum value is ∼0.3 and varies very little between seeds of the same species. Following the calculations of Thompson *et al.* (1993), we calculated the seed shape index as the variance of seed length, width and thickness. To prevent seed size from affecting the index, we first standardized the three dimensions by scaling them relative to seed length, which was set to 1.

To study the association of TSM and seed shape index with seed persistence, we used seed persistence as a binary dependent variable (transient versus persistent). Following the approach of Gioria *et al.* (2020) and Wang *et al.* (2024) for example, species with SBPI values of zero were treated as having transient seeds, while species with an SBPI higher than zero were treated as having persistent seeds, because an SBPI higher than zero indicates that there was at least one study from Hungary finding a persistent seed bank for the species in question.

To correct for the phylogenetic autocorrelation in our data, we used phylogenetic comparative methods. We used the Daphne phylogenetic tree, which is a dated, ultrametric tree of Central European vascular plants encompassing 4685 species (Durka and Michalski, 2012). Of the species in our dataset, 96.5 % were represented on the Daphne tree; therefore, for the phylogenetically informed analyses we reduced our dataset to these 368 species and pruned the tree accordingly. The Daphne tree contains polytomies, which we resolved into a binary (bifurcating) tree using the function multi2di from the R package APE (Paradis *et al.*, 2004).

To analyse the relationship of TSM, seed shape index and their interaction with seed persistence as a binary variable (persistent versus transient), we applied phylogenetic logistic regressions (Ives and Garland, 2010) with the function phyloglm provided in the R package phylolm (Ho and An, 2014). We used the logistic_MPLE method with 1000 independent bootstrap replicates. Phyloglm uses *α* to quantify the phylogenetic signal, with smaller *α* values indicating a stronger phylogenetic signal. We also applied standard logistic regressions and compared the standard and phylogenetic models based on the Akaike information criterion (AIC). Because TSM and the seed shape index are on very different scales (TSM ranged from 0.004 to 56.14 g, while the seed shape index ranged from 0.00013 to 0.29329), we used *z*-scoring prior to the analysis to standardize them so that both variables have a mean of 0 and a standard deviation of 1, which also makes the coefficients and odds ratios more comparable.

We also used phylogenetic logistic regressions to analyse the relationship of TSM and seed shape index with seed persistence in four plant functional groups defined by two complementary classification aspects: growth form (forbs versus graminoids) and lifespan (perennial versus short-lived). Thus, the first two groups (forbs and graminoids) and the latter two groups (perennials and short-lived species) refer to the same set of species but classified according to different criteria. Life form was assigned to species according to Sonkoly *et al.* (2023) and we considered therophyte and hemitherophyte species to be short-lived (129 species after reducing our dataset to species also included in the Daphne tree). Species in other life form categories were considered perennial (239 species). Species in the Cyperaceae, Juncaceae and Poaceae families were considered graminoids (87 species) and all other herbaceous species were considered to be forbs (283 species). We compared the TSM and the seed shape index of persistent versus transient species using the function phylANOVA in the phytools package (Revell, 2024), which performs a simulation-based phylogenetic ANOVA (Garland *et al.*, 1993). As some *P*-values were close to the 0.05 significance threshold, we used 10 000 simulations (nsim = 10 000) to ensure sufficient precision.

To test how TSM and seed shape index are related to each other across all species and in different functional groups, we performed phylogenetic generalized least squares (PGLS) regressions with TSM as dependent and seed shape index as explanatory variable. Based on the recommendation of Chen *et al.* (2023), we calculated Pagel’s *λ* (a widely used phylogenetic signal metric; Pagel, 1999) for both traits and used the one with a higher Pagel’s *λ* as the dependent variable. We implemented PGLS regressions using the R packages APE (Paradis *et al.*, 2004) and nlme (Pinheiro *et al.*, 2014). Pagel’s *λ* was calculated using the phylosig function in the phytools R package (Revell, 2024). All analyses were carried out in an R environment (version 4.3.2; R Core Team, 2023). Nomenclature follows Euro + Med PlantBase (Greuter *et al.,* 2024, http://www.europlusmed.org).

## RESULTS

After the exclusion of aquatic and woody species, and reducing our dataset to species also included in the Daphne tree, we performed the analyses with a dataset containing data on 368 species (Supplementary Data Table S1). In this reduced dataset, TSM ranged from 0.004 g (*Gnaphalium uliginosum* and *Sagina procumbens*) to 56.14 g (*Iris pseudacorus*) while seed shape index ranged from 0.00013 (*Vicia angustifolia*) to 0.29329 (*Stipa borysthenica*).

**Fig. 1.**
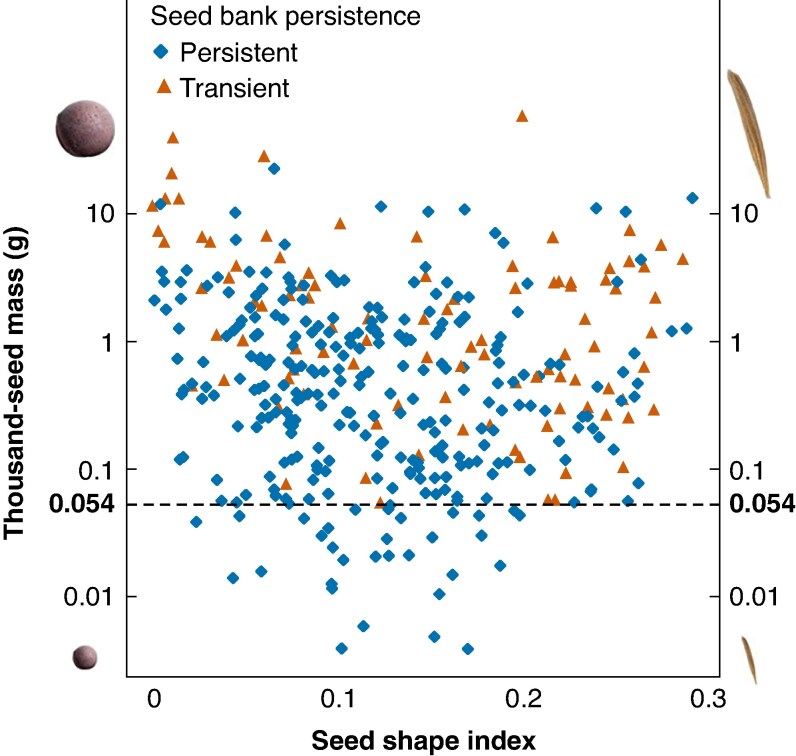
Relationship between seed mass, seed shape and seed bank persistence in 392 herbaceous species of the Pannonian flora. The dashed line indicates the thousand-seed mass value (0.054 g) below which all studies species have persistent seed banks. Note that the *y*-axis is on a logarithmic scale. Seeds of contrasting sizes and shapes are depicted in the four corners of the graph for easier interpretation.

Across all the species, the phylogenetic logistic regression model estimated a low phylogenetic correlation parameter (*α* = 0.095), indicating a strong phylogenetic signal in seed persistence, corroborating that correcting for phylogenetic non-independence in the model was necessary. Accounting for phylogenetic non-independence, we found that both TSM and seed shape index were significantly negatively related to seed persistence, with TSM having a stronger relationship than seed shape index (Table 1). The interaction term was also significant, indicating that at higher seed shape index values the relationship of TSM and persistence becomes less negative (Fig. 1). The threshold TSM was found to be 0.054 g, meaning that all studied species with a TSM lower than this had a persistent seed bank (Fig. 1). Model comparison based on the AIC also indicated that the phylogenetic logistic regression provided a better fit than the standard logistic regression (*Δ*AIC = 3.476, AIC = 375.06 and AIC = 378.53, respectively), validating the use of phylogenetic correction in the analysis.

**Table 1.** Results of the phylogenetic logistic regression model testing the effects of thousand-seed mass (TSM), seed shape index and their interaction on the seed bank persistence of all herbaceous species.

	Coefficient (estimate)	Odds ratio	95 % confidence interval (odds ratio)	*P*-value
TSM	−0.649	0.522	−1.097 to −0.346	*<0*.*001*
Seed shape index	−0.487	0.615	−0.751 to −0.234	*<0*.*001*
TSM × seed shape index	0.455	1.576	0.121 to 0.782	*0*.*003*

TSM and seed shape index values were scaled before the analysis (*z*-scoring).

Significant differences are marked with italics.

**Fig. 2.**
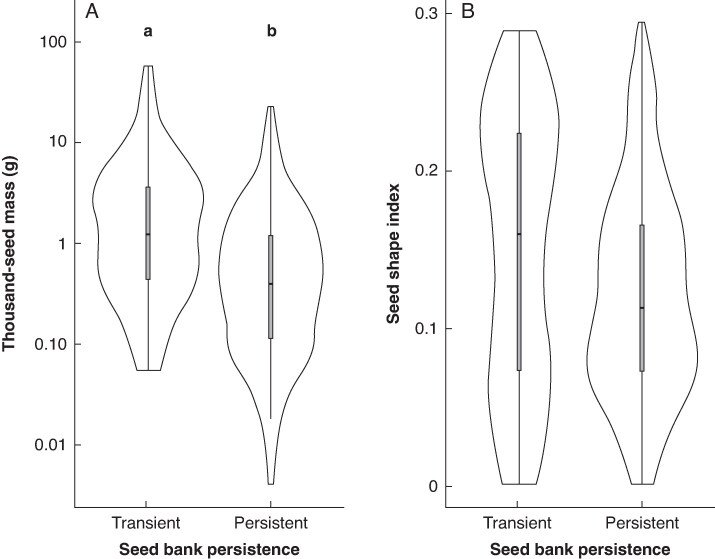
Thousand-seed mass (A) and seed shape index (B) of species with transient versus persistent seed banks. Data are for all species included in the analysis. Different letters above the bars denote significant differences (phylogenetic ANOVAs). Note that in (A) the *y*-axis is on a logarithmic scale.

By analysing the seed mass of species using phylogenetic ANOVA, we found that the seed mass of species with transient seeds was significantly higher than that of species with persistent seeds after correcting for phylogenetic non-independence (*F* = 24.226, *P* = 0.0016; Fig. 2). However, phylogenetic ANOVA did not show a significant difference between the seed shape index of species with transient and persistent seeds (*F* = 10.384, *P* = 0.0557).

**Fig. 3.**
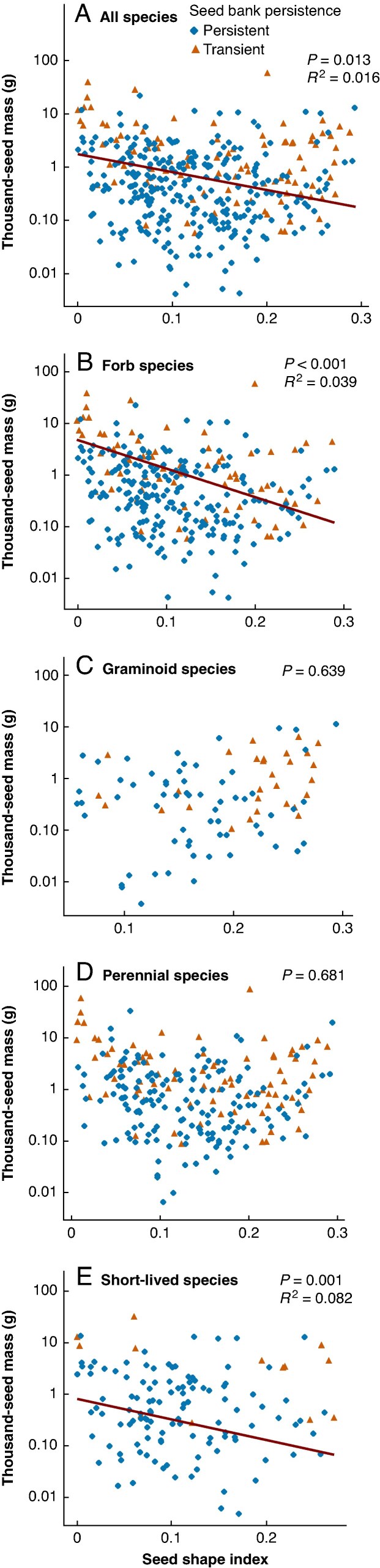
Relationship between seed mass, seed shape and seed bank persistence in different functional groups accounting for phylogeny: (A) all species; (B) forb species; (C) graminoid species; (D) perennial species; (E) short-lived species. The relationships between seed mass and seed shape were tested using PGLS regressions. Red lines are the PGLS regression lines.

To assess whether the effect of TSM and seed shape index on seed persistence is consistent across functional groups, we also studied the relationship separately in four functional groups (Fig. 3). In the case of forb species, the phylogenetic logistic regression model estimated a low phylogenetic correlation parameter (*α*=0.039), indicating a strong phylogenetic signal in seed persistence in forb species as well. The model indicated that in forb species, TSM, seed shape index and their interaction had a significant negative association with seed persistence after correcting for phylogenetic non-independence (Table 2). The phylogenetic logistic regression model for forb species provided a slightly better fit to the data than the standard logistic regression (*Δ*AIC = 0.68, AIC = 280.24 and AIC = 280.92, respectively). Phylogenetic ANOVA indicated that the seed mass of species with transient seeds was significantly higher than the seed mass of species with persistent seeds in the forb functional group as well (*F* = 24.870, *P* = 0.0008), but the seed shape index did not differ significantly between the two groups in the case of forb species (*F* = 1.365385, *P* = 0.466; Supplementary Data Fig. S1).

**Table 2.** Results of phylogenetic logistic regression models testing the effects of thousand-seed mass (TSM), seed shape index and their interaction on the seed bank persistence of species in different functional groups.

	Coefficient (estimate)	Odds ratio	95 % confidence interval (odds ratio)	*P*-value
Forb species (*n* = 301)				
TSM	−0.726	0.484	−1.276 to −0.299	*<0*.*001*
Seed shape index	−0.424	0.654	−0.727 to −0.076	*0*.*015*
TSM × seed shape index	0.461	1.585	0.044 to 0.921	*0*.*005*
Graminoid species (*n* = 91)				
TSM	−0.405	0.667	−1.163 to 0.254	0.259
Seed shape index	−0.911	0.402	−1.678 to −0.242	*0*.*002*
TSM × seed shape index	0.443	1.558	−0.058 to 1.100	0.074
Perennial species (*n* = 256)				
TSM	−0.622	0.537	−1.288 to −0.243	*0*.*012*
Seed shape index	−0.307	0.736	−0.612 to 0.006	0.056
TSM × seed shape index	−0.413	1.511	−0.024 to 0.920	0.054
Short-lived species (*n* = 136)				
TSM	−0.907	0.404	−1.549 to 0.000	*0*.*007*
Seed shape index	−0.895	0.409	−1.589 to −0.117	*0*.*015*
TSM × seed shape index	0.534	1.706	0.000 to 1.214	*0*.*039*

TSM and seed shape index values were scaled before the analysis (*z*-scoring).

Significant differences are marked with italics.

In the case of graminoid species, the phylogenetic logistic regression model also estimated a low phylogenetic correlation parameter (*α* = 0.011), and therefore a strong phylogenetic signal in seed persistence. The model indicated that in graminoid species only seed shape index was significantly associated with seed persistence (Table 2). In this case, using a phylogenetic logistic regression did not result in better model fit compared with a standard logistic regression (*Δ*AIC = 3.8501, AIC = 105.957 and AIC = 102.106, respectively). In line with the results of the phylogenetic logistic regression, phylogenetic ANOVA indicated that in graminoid species seed mass was not significantly different between species with transient seeds and species with persistent seeds (*F* = 1.906, *P* = 0.547), while the seed shape index of species with transient seeds was significantly higher than that of species with persistent seeds (*F* = 14.095, *P* = 0.016; Supplementary Data Fig. S1).

The phylogenetic logistic regression model estimated a low phylogenetic correlation parameter (α= 0.077) in the case of perennial species as well, therefore a strong phylogenetic signal in seed persistence. In perennial species only seed mass was significantly associated with seed persistence (Table 2). The phylogenetic logistic regression model for perennial species provided a better fit to the data compared with the standard logistic regression (*Δ*AIC = 4.609, AIC = 329.893 and AIC = 334.502, respectively). The phylogenetic ANOVA indicated that in perennial species the seed mass of species with transient seeds was significantly higher than that of species with persistent seeds (*F* = 16.063, *P* = 0.002), while seed shape index did not differ significantly between the two groups (*F* = 2.797, *P* = 0.193; Supplementary Data Fig. S1), which is also in line with the results of the phylogenetic logistic regression.

In the case of short-lived species, the phylogenetic logistic regression model also estimated a low phylogenetic correlation parameter (*α* = 0.060), indicating a strong phylogenetic signal in seed persistence in this functional group as well. In the subset of short-lived species, seed mass, seed shape index and their interaction were significantly negatively associated with seed persistence (Table 2). In this case, using a phylogenetic logistic regression model provided a slightly better model fit than the standard logistic regression (*Δ*AIC = 0.219, AIC = 69.489 and AIC = 69.708, respectively). The phylogenetic ANOVA indicated that, in the short-lived group, species with transient seeds had a significantly higher seed mass than species with persistent seeds (*F* = 22.178, *P* = 0.0003), but seed shape index did not differ significantly between the two groups (*F* = 5.526, *P* = 0.093; Supplementary Data Fig. S1).

To test whether TSM and seed shape index are related to each other, we used PGLS regression models. Across all species, we found a very weak negative relationship between TSM and seed shape index after accounting for phylogenetic non-independence (Fig. 3A). Similarly weak but significant negative relationships were found between these two variables in the forb and short-lived functional groups (Fig. 3B, E), while there was no significant relationship in the case of graminoid species (Fig. 3C) and perennial species (Fig. 3D).

## DISCUSSION

In line with the findings of several previous studies (e.g. Thompson *et al.*, 1993; Funes *et al.*, 1999; Zhao *et al.*, 2011), accounting for phylogenetic non-independence, we found that although the relationships are relatively weak, both seed mass and seed shape are significantly negatively related to seed persistence in 368 herbaceous species of the Pannonian flora. However, seed shape appears to be less closely associated with seed persistence in this region. Several previous studies have found that only seed size is significantly related to seed persistence in various regions of the world (e.g. Bekker *et al.*, 1998; Peco *et al.*, 2003; Yu *et al.*, 2007; Wang *et al.*, 2011). Therefore, it seems to be a rather common trend that seed shape is less important than seed size in determining seed persistence. However, there are also results implying that only seed shape significantly affects seed persistence (McDonald *et al.*, 1996).

Seeds with a TSM <0.054 g were all persistent, implying that in the Pannonian flora all seeds with a mass below this threshold value may be considered persistent with reasonable certainty. However, many persistent seeds were relatively large, and therefore an upper threshold above which all seeds could be considered transient cannot be identified. Similarly, although spherical seeds were found to be more likely to be persistent, many persistent seeds were markedly non-spherical, in line with the findings of Moles *et al.* (2000).

Our results agree with the notion that seed persistence cannot be reliably predicted based only on seed mass (Gioria *et al.*, 2020); seed shape or perhaps other seed characteristics such as seed coat thickness, dormancy mechanisms or nutrient reserves may also need to be considered (e.g. Davis *et al.*, 2016; Zalamea *et al.*, 2018). Using seed bank persistence data provided by Thompson *et al.* (1997) and seed mass data measured in the Pannonian region, Csontos and Tamás (2003) demonstrated that the proportion of transient species is increasing with increasing seed mass in the Pannonian flora. This indicates that the relationship between seed size and persistence is the same in the Pannonian flora as in most other regions where it was studied, but seed shape has not been considered in this analysis. Moreover, the analysed seed bank persistence data originated from different regions, which may have a confounding effect.

Although Wang *et al.* (2024) found no interaction between seed mass and seed shape, the interaction between them was significant in our analysis encompassing all species. However, in contrast to the analysis of Wang *et al.* (2024), our analysis only included herbaceous non-aquatic species. The negative interaction term indicated that at higher seed shape index values the effect of TSM on persistence is less negative, which may be the result of the fact that above a seed shape index of ∼0.2 there were no species with very small seeds in the dataset. This negative interaction may not exist in floras containing several species with very small, non-spherical seeds.

Seed mass and shape may be related to seed persistence due to a number of reasons. The size and shape of seeds presumably affect their ability to move towards deeper soil layers (e.g. Bekker *et al.*, 1998; Schmiede *et al.*, 2009). For example, there are studies suggesting that large and elongated seeds are less likely to be buried by the activity of soil biota (Thompson *et al.*, 1994; Bernhardt, 1995). One theory is that the correlation may be due to this tendency of small and isodiametric seeds to quickly become buried in the soil, because being able to persist until a disturbance brings them to the soil surface again may commonly be necessary for these seeds (Moles *et al.*, 2000). As seeds experience higher rates of predation on the soil surface compared with when they are buried (Hulme, 1998; Jacob *et al.*, 2006), it can also be hypothesized that large and elongated or flattened seeds with slow soil penetration cannot escape predation by quickly being buried in the soil (Hulme and Borelli, 1999). Therefore, for these species it may be less advantageous to build a persistent seed bank. Buried seeds are also less exposed to germination-stimulating temperature fluctuations and light (Fenner and Thompson, 2005). Larger seeds are generally able to germinate from deeper soil layers (Grundy *et al.*, 2003; Sonkoly *et al.*, 2020), while soil burial typically hinders the germination of small seeds, because they are more likely to have a light requirement for germination (Milberg *et al.*, 2000). This means that they are more likely to remain ungerminated once they are buried, providing them an opportunity to persist in the soil.

Whether the relationship between seed persistence and the size and shape of seeds varies between different plant functional groups has also not been studied previously. For example, it is known that to ensure survival during periods of unfavourable environmental conditions, short-lived species depend more strongly on persistent seeds than perennial species (Meyer *et al.*, 2006; Scott *et al.*, 2010). Accordingly, short-lived plant species tend to have more persistent seeds than perennials (Gioria *et al.*, 2020), which are typically also smaller-sized than the seeds of perennial species (Thompson *et al.*, 1998; Wang *et al.*, 2011). A short lifespan can also be associated with persistent seeds through the disturbance regime of the habitat. The proportion of short-lived species is higher in disturbed habitats than in relatively undisturbed ones, as disturbance can lead to changes in plant community composition in favour of species with rapid growth and with a resource-acquisitive strategy (Smith *et al.*, 2022). As ensuring the survival of the population in disturbed habitats requires the formation of a persistent seed bank (Fenner and Thompson, 2005), the relationship between seed traits and seed persistence may not be the same in short-lived and perennial species. In this context, it has already been demonstrated that different plant functional groups such as annuals and perennials can have contrasting relationships between seed size and several other factors, such as competitive ability and seed production (Coomes and Grubb, 2003). Seed bank types can also be contrasting in forb and graminoid species even within the same habitat type (Bertiller and Aloia, 1997), and the relationship between habitat characteristics and the proportion of species with persistent seeds can differ significantly in forb and graminoid species (Zeiter *et al.*, 2013). Moreover, the ability of a species’ seeds to persist is also related to phylogeny (Gioria *et al.*, 2020).

Based on the above considerations, the relationship between seed traits and seed persistence may vary considerably across different plant functional groups, and our findings confirm this assumption. Accounting for phylogenetic non-independence, we found that both seed mass and seed shape were associated with seed persistence across all species and in the forb and short-lived groups, with seed mass having a stronger relationship with seed persistence in all the above groups. In contrast to this, only seed shape was related to seed persistence in the case of graminoid species, and only seed mass in the case of the perennial group. Although the relationships are relatively weak, these results suggest that there are different effects at play in forb and in graminoid species. Wang *et al.* (2024) found that the relationship of seed mass and shape with seed persistence is not consistent across phylogenetic clades. According to their findings, both seed mass and shape affect persistence in Poales, but only seed mass affects persistence in Asterales and Lamiales, while no significant effect was detected in Fabales and Caryophyllales. Although the nature of the relationship they found in Poales is not the same as what we found in the graminoid group (which consisted mainly of Poales species), their findings are consistent with our results in the sense that the relationship between seed traits and seed persistence in graminoids is different from the relationship seen in other species. Moreover, the relationship of these variables may also differ between short-lived and perennial graminoid species. Taken together, these findings suggest that graminoids exhibit distinct relationships and might have to be treated and analysed separately from other species to disentangle the complex relationships between seed traits and seed bank persistence.

If the relationship of seed size and shape with seed persistence is not consistent across different plant functional groups, it may be because they are differently related to each other in different functional groups, which could at least partially explain inconsistencies. However, most previous studies about the influence of seed size and shape on seed persistence have not assessed whether and how seed size and shape themselves are correlated (e.g. Moles *et al.*, 2000; Peco *et al.*, 2003; Wang *et al.*, 2024), leaving this question unresolved. To our knowledge, the study of Zhao *et al.* (2011) is the only exception. They studied 141 species of sand grasslands in northern China and found a slight tendency of bigger seeds to be more spherical, but the relationship was not significant. We addressed this knowledge gap by examining the relationship between seed size and shape within and across plant functional groups, while also accounting for phylogeny. We found a weak negative correlation between seed mass and seed shape index in all species and in the functional groups of forbs and short-lived species, while, after accounting for phylogeny, there was no significant correlation in graminoid and perennial species. In graminoids, seed shape may counteract or modify the commonly observed effect of seed mass on persistence, making seed mass a non-significant predictor of persistence in this group. In the studied 91 graminoid species of the Pannonian flora, small-seeded species were generally found to have more spherical seeds (e.g. *Juncus* or *Agrostis* species), while larger graminoid seeds are typically more elongated (e.g. *Stipa* or *Bromus* species). Csontos and Kalapos (2013) also found that larger seeds tend to be less isodiametric in 137 grass species of the Pannonian flora with C3 photosynthesis. This trend therefore seems to be quite obvious in the Pannonian flora, but this might not be the case in other regions of the world, which could also cause a different relationship between seed size and shape and seed persistence in the graminoids of other floras. A possible future direction would therefore be to test whether the seed persistence of graminoid species is also influenced solely by seed shape in other floras as well.

### Conclusions

Persistent seed banks have a key role in community resilience and in the maintenance of biodiversity through space and time; therefore, enhancing our knowledge of the formation of persistent seed banks is vitally important. Our results contribute to a better understanding of the factors linked to seed persistence in the soil. We found that, similarly to the floras of several other regions, both seed size and seed shape are significantly related to seed persistence in the Pannonian flora, with seed shape having a less strong influence. By analysing the relationship between these factors in different plant functional groups separately, we also revealed that graminoid species show distinct relationships. Therefore, although the general trend found in our study is consistent with most previous analyses of seed size, seed shape and persistence, our more detailed results regarding different plant functional groups suggest that detailed analyses are necessary in the floras of other regions as well, and future studies of this relationship may need to treat and analyse graminoid species separately.

## Supplementary Material

mcaf322_Supplementary_Data

## Data Availability

All the data generated for and used in this study are available in Supplementary Data Table S1.

## SUPPLEMENTARY DATA

Supplementary data are available at *Annals of Botany* online and consist of the following. Table S1: the generated dataset containing the Seed Bank Persistence Index, thousand-seed mass, seed length, seed width, seed thickness, seed shape index and further information about the studied 392 species of the Pannonian flora. Figure S1: thousand-seed mass and seed shape index of transient and persistent species in different plant functional groups.
