# Supplementary material for: Inconsistent relationships detected between seed size, shape and persistence for different plant functional groups in the Pannonian flora
Source: Ann Bot. 2025 Dec 12;137(4):1026–35. doi: 10.1093/aob/mcaf322 (PMC13095884; doi:10.1093/aob/mcaf322)
Supplement: mcaf322_Supplementary_Data [file mcaf322_supplementary_data.zip › Toro-Szijgyarto.et.al.-Supplementaty-FigureS1-revised.pdf]

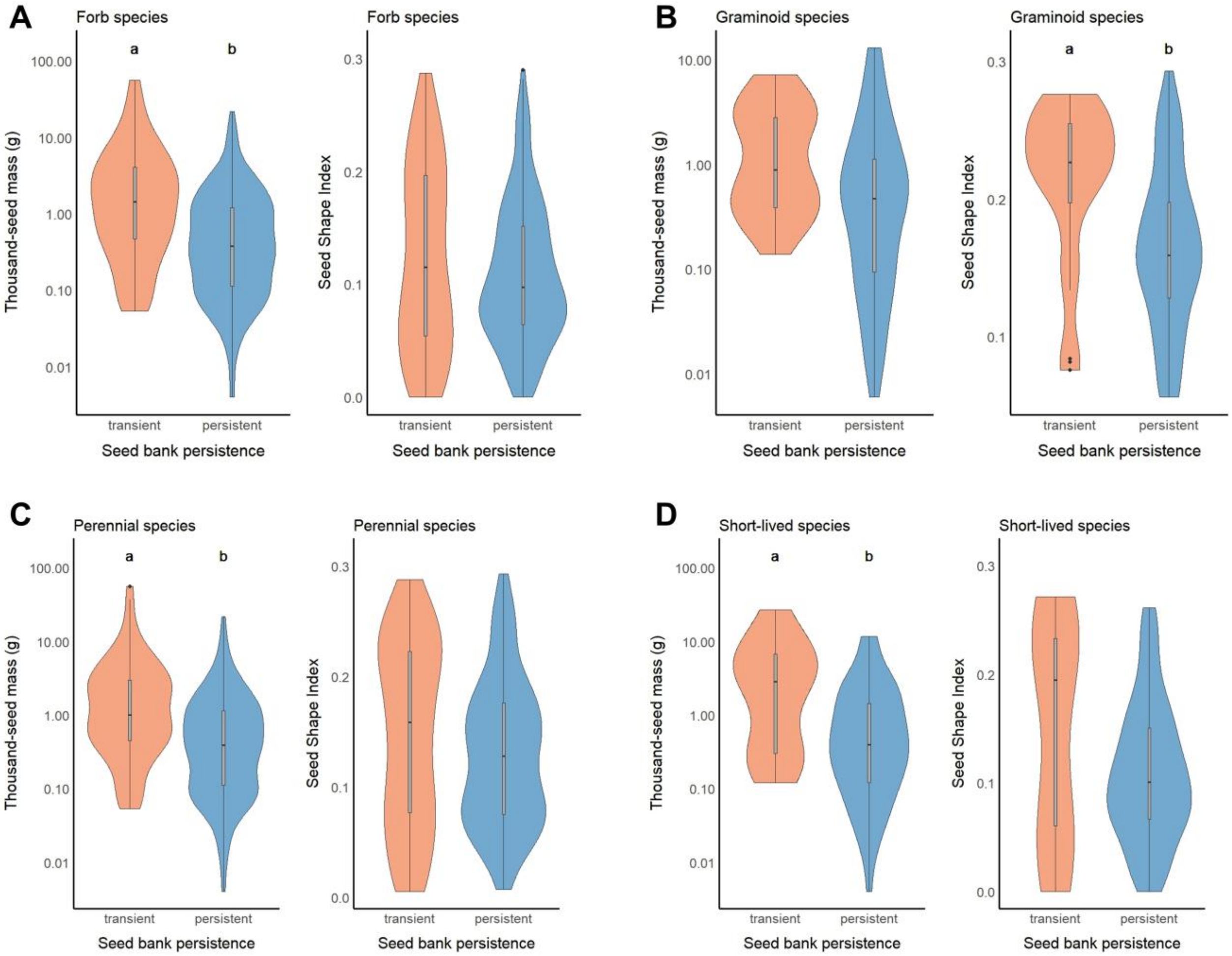

Figure S1. The thousand-seed mass and Seed Shape Index of transient and persistent species in different plant functional groups. Significant differences based on phylogenetic ANOVAs are indicated by different letters above the bars. A – forb species; B – graminoid species; C – perennial species; D – short-lived species.
